# Chatbots for Well-Being: Exploring the Impact of Artificial Intelligence on Mood Enhancement and Mental Health

**DOI:** 10.1192/j.eurpsy.2024.1143

**Published:** 2024-08-27

**Authors:** R. M. Lopes, A. F. Silva, A. C. A. Rodrigues, V. Melo

**Affiliations:** Psychiatry and Mental Health Department, Centro Hospitalar Médio Tejo, Tomar, Portugal

## Abstract

**Introduction:**

Over the past few years, Psychiatry has undergone a significant transformation with the integration of Artificial Intelligence (AI). This shift has been driven by the increasing demand for mental health services, as well as advances in AI technology. AI analyzes extensive datasets, including text, voice, and behavioral data, aiding in mental health diagnosis and treatment. Consequently, a range of AI-based interventions has been developed, including chatbots, virtual therapists and apps featuring cognitive-behavioral therapy (CBT) modules. Notably, chatbots, as conversational agents, have emerged as valuable tools, assisting users in monitoring emotions and providing evidence-based resources, well-being support, psychoeducation and adaptive coping strategies.

**Objectives:**

This study aims to investigate the impact of AI chatbots on improving mental health, evaluate their strengths and weaknesses and explore their potential for early detection and intervention in mental health issues.

**Methods:**

A literature review was conducted through PubMed and Google Scholar databases, using keywords ‘artificial intelligence’, ‘chatbot’ and ‘mental health’. The selection focused on the most relevant articles published between January 2021 and September 2023.

**Results:**

Mental health chatbots are highly personalized, with a primary focus on addressing issues such as depression or anxiety within specific clinical population groups. Through the integration of Natural Language Processing (NLP) techniques and rule-based AI algorithms, these chatbots closely simulate human interactions and effectively instruct users in therapeutic techniques. While chatbots integrating CBT principles have gained widespread use and extensive research attention, some also incorporate alternative therapeutic approaches, including dialectical behavior therapy, motivational interviewing, acceptance and commitment therapy, positive psychology or mindfulness-based stress reduction. AI chatbots provide substantial advantages in terms of accessibility, cost-effectiveness and improved access to mental health support services. Nonetheless, they also exhibit limitations, including the absence of human connection, limited expertise, potential for misdiagnosis, privacy concerns, risk of bias and limitations in risk assessment accuracy.

**Conclusions:**

AI-based chatbots hold the potential to enhance patient outcomes by enabling early detection and intervention in mental health issues. However, their implementation in mental health should be approached with caution. Further studies are essential to thoroughly evaluate their effectiveness and safety.

**Disclosure of Interest:**

None Declared

